# Bayesian reconstruction of memories stored in neural networks from their connectivity

**DOI:** 10.1371/journal.pcbi.1010813

**Published:** 2023-01-30

**Authors:** Sebastian Goldt, Florent Krzakala, Lenka Zdeborová, Nicolas Brunel

**Affiliations:** 1 International School of Advanced Studies (SISSA), Trieste, Italy; 2 IdePHICS laboratory, Ecole Polytechnique Fédérale de Lausanne (EPFL), Switzerland; 3 SPOC laboratory, Ecole Polytechnique Fédérale de Lausanne (EPFL), Switzerland; 4 Department of Neurobiology, Duke University, Durham, North Carolina, United States of America; 5 Department of Physics, Duke University, Durham, North Carolina, United States of America; Université Paris Descartes, Centre National de la Recherche Scientifique, FRANCE

## Abstract

The advent of comprehensive synaptic wiring diagrams of large neural circuits has created the field of connectomics and given rise to a number of open research questions. One such question is whether it is possible to reconstruct the information stored in a recurrent network of neurons, given its synaptic connectivity matrix. Here, we address this question by determining when solving such an inference problem is theoretically possible in specific attractor network models and by providing a practical algorithm to do so. The algorithm builds on ideas from statistical physics to perform approximate Bayesian inference and is amenable to exact analysis. We study its performance on three different models, compare the algorithm to standard algorithms such as PCA, and explore the limitations of reconstructing stored patterns from synaptic connectivity.

## Author summary

One of the central hypothesis of neuroscience is that memories are stored in synaptic connectivity. Theoretical models show how large numbers of memories can be stored in recurrent neural circuits thanks to synaptic plasticity mechanisms. Recent advances in serial block-face electron microscopy, and machine learning methods, are making it possible to fully reconstruct the synaptic connectivity of neuronal circuits of increasingly large volumes. Here, we ask the question to what extent it is possible to reconstruct memories stored in a neural circuit from the knowledge of its synaptic connectivity. We present an approximate Bayesian inference algorithm, and study its properties on specific attractor network models.

## Introduction

Comprehensive synaptic wiring diagrams or “connectomes” provide a detailed map of all the neurons and their interconnections in a brain region or even an entire organism. Since the connectome of the nematode C. elegans was obtained using electron microscopy methods in 1986 [1], methods for data acquisition and analysis have both been scaled up and improved significantly. Today, it has become possible to provide connectomes of much more complex systems such as various *Drosophila melanogaster* circuits [2, 3], or even a large part of its brain [4, 5]; the olfactory bulb of zebrafish [6]; and various pieces of the rodent retina [7–9], hippocampus [10], and cortex [11–16]. While there still remain a number of formidable challenges on the way to the complete connectome of a mammal or even human brain [17], the data sets available today already give rise to a number of intriguing questions. At the same time, it is becoming increasingly clear that new quantitative methods must be developed to fully exploit the new troves of data that connectomics provides [18].

Here, we focus on local neural networks that store information in their synaptic connectivity. It has been hypothesised that cortical networks are optimised for this task, thanks to their extensive recurrent synaptic connectivity [19]. A popular model for these networks are attractor neural networks such as the Hopfield model [20] and various generalisations [21–24], in which memories are stored as attractor states of the dynamics. These attractor states represent learned internal representations of external stimuli that have been presented repeatedly to the network during training, inducing changes in synaptic weights of the network. One natural question to ask is then: given the knowledge of the synaptic connections between neurons in a recurrent neural network, can we reconstruct the patterns of activity that were stored in this network in the first place?

In this paper, we first give a mathematical formulation of this problem in terms of a Bayesian inference problem. The Bayesian approach has the advantage of providing a natural way to handle the uncertainty associated with estimating a large number of parameters from a large number of noisy observations, *i.e.* the reconstruction of the original stimuli from the strength of the synapses in the networks in the present case. Modelling the noise is crucial in this problem, as we cannot expect the synaptic strengths reported in connectomes to be more than rough estimates. We use tools from statistical physics to both determine when solving this inference problem is theoretically possible in a model setting, and to provide a practical algorithm to do so. We analyse the performance of the algorithm in detail on a variety of different problems, and we invite the reader to download our reference implementation of the algorithm on GitHub and to use and extend it.

### The task: Reconstructing memories from network connectivity

#### The network model

We analyse a variant of the celebrated Hopfield model [20] for a recurrent neural network composed of *N* interacting neurons with state *s*_*i*_, *i* = 1, …, *N*. The network is fully-connected with symmetric, bidirectional connections that have a scalar weight $J_{ij}=J_{ji}\in R$. The neurons update their state at iteration *k* + 1 sequentially according to

$$\begin{array}{c} s_{i}^{k+1}=g\left(a_{i}^{k}\right), \end{array}$$ (1)

where *g*(⋅) is some non-linear activation function and $a_{i}^{k}=\sum_{j\neq i}\;J_{ij}s_{j}^{k}$ is the total synaptic input of the *i*th neuron.

The network stores *P* fixed patterns or memories, which are *N*-dimensional vectors that we collect in the matrix $X^{*}\in X^{P\times N}$. We write $X_{\mu ,:}^{*}$ for the *μ*th pattern stored in the network, and X denotes the set of values that pattern entries can take, *e.g.* X={±1} for binary patterns. Note that these patterns correspond to deviations of neuronal activity from its mean, and not neuronal activity itself, which is constrained to be non-negative. This subtraction of mean activity is expected to be performed by the plasticity rule operating at the synapse (using a ‘covariance rule’, see e.g. [25]). In the case of binary patterns, *X*_*μ*,*i*_ = 1 means a neuron is active in a given pattern, while *X*_*μ*,*i*_ = −1 means a neuron is inactive. We also assume that *X*_*μ*,*i*_ are i.i.d. random variables, i.e. that stored patterns are uncorrelated. Patterns are stored in the network by choosing its weights *J*_*ij*_ such that the patterns $X_{\mu ,:}^{*}$ become fixed points of its dynamics.

We study this model in the thermodynamic limit *N* → ∞, while keeping the number of patterns *P* of order 1. This scaling makes the resulting weight matrix of the Hopfield model a low-rank matrix. Low rank matrices have played an important role in neuroscience in recent years, in particular for the modelling of recurrent networks [26–29]. Hence, methods to estimate them from data in a principled way can help connect these theories with experimental data.

#### The learning rule

A classic idea for choosing the weights, or the connectivity structure of the network $J=\left[J_{ij}\right]\in R^{N\times N}$, is to choose the weights proportional to the empirical correlation of the patterns, $W_{ij}∼\sum_{\mu }X_{\mu ,i}^{*}X_{\mu ,j}^{*}$. This prescription is also known as the Hebb rule [30] and can be written more compactly as

$$\begin{array}{c} J=W=\frac{1}{\sqrt{N}}X^{*}{\left(X^{*}\right)}^{⊤}, \end{array}$$ (2)

where (X*)^⊤^ is the transpose of X* and ***W*** is thus the empirical correlation matrix of the patterns, assuming that the means of the patterns are zero, as we will do throughout this work, and the choice of the $1/\sqrt{N}$ scaling is explained below. With binary neurons *s*_*i*_(*t*) = ±1, this model corresponds to the celebrated Hopfield model [20]. In this model, the network exhibits fixed point attractors close to the stored memories, provided the number of stored patterns *P* is smaller than *α*_*c*_*N* where *α*_*c*_ ∼ 0.14 [31].

The connectivity matrix Eq (2) has a number of unrealistic features that makes it inadequate for the problem we are interested in here: (i) The network is fully connected, at odds with neuronal networks in the brain; (ii) Synapses are not sign-constrained, while synapses in the brain are either excitatory (i.e. non-negative) or inhibitory (i.e. non-positive). A minimal model that satisfies both requirements is the **rectified Hopfield model**

$$\begin{array}{c} J_{ij}=\Phi \left(W_{ij}-\tau +\zeta_{ij}\right)\geq 0 \end{array}$$ (3)

where *τ* > 0, *ζ*_*ij*_ is a noise term (see below), and we choose *Φ*(*x*) = max(0, *x*). This choice ensures that weights are non-negative, effectively yielding a model of a network of excitatory neurons. This is consistent with the hypothesis that information storage occurs primarily in excitatory-to-excitatory synapses, while the job of inhibitory neurons is primarily to control the level of activity in the excitatory network. This view is consistent with a number of studies [32, 33] but has been challenged by others [34].

The noise term *ζ*_*ij*_ is taken to be a symmetric random Gaussian matrix, i.e. for *i* < *j*, *ζ*_*ij*_s are i.i.d. random Gaussian variables with mean zero and standard deviation *ν*, and *ζ*_*ji*_ = *ζ*_*ij*_. The scalar parameter *τ* controls the connection probability in the network,

$$\begin{array}{c} p_{C}=p\left(J_{ij}>0\right)=\frac{1}{2}\text{erfc}(\frac{\tau }{\sqrt{2}\nu }) \end{array}$$ (4)

to leading order as *N* → ∞. In particular, the network becomes sparse in the large *τ* limit. Since *P* ∼ *O*(1), the weights obtained from Eq (2) will have variance 1/*N*, while the noise has variance *ν* of order 1. The model we study is related to a family of connectivity matrices studied by Sompolinsky [35], and bears similarities with a model recently proposed by Mongillo *et al.* [34].

In brain networks, connectivity matrices are not symmetric. However, if we assume that the weights depend on the stimuli only via the symmetric matrix X*(X*)^⊤^, these asymmetries will be due to the different sources of noise in the learning process, and the fact that connectomic reconstructions will give us at best an approximation of true synaptic weights (see Discussion). For the purpose of analysing quantities derived from the symmetric matrix X*(X*)^⊤^, as we will do here, we can hence symmetrise the matrix ***J***, or equivalently focus on the case where the noise matrix *ζ*_*ij*_ is symmetric.

#### A note on the 1N scaling

Our choice of scaling in Eq (2) is made with reconstructing the patterns in mind and follows from random matrix theory. Our model for the connectivity matrix is related to the spiked Wigner model [36, 37], where a random matrix ***M*** is constructed as *M*_*ij*_ = *βu*_*i*_*u*_*j*_ + *ζ*_*ij*_, with *ζ*_*ij*_ as above. The matrix ***M*** is hence a rank-one perturbation of a random matrix with elements drawn i.i.d. from the normal distribution. The task is to reconstruct the vector ***u***, whose elements are of order 1, from the matrix ***M***. Intuitively, we want to put ourselves in the “interesting” regime, where reconstruction of the *N* entries of ***u*** from the $O(N^{2})$ elements of ***M*** is neither trivially easy nor impossible. A classic analysis of this model using random matrix theory [36, 38] reveals that this “interesting” regime is characterised by a phase transition in the overlap between the leading eigenvector of ***M*** and the vector ***u***, which occurs precisely for a signal-to-noise ratio *β* that scales as 1N. We thus choose the same scaling for our non-linear matrix model (3).

#### Reconstructing vs retrieving the memories

We emphasise that our focus in this paper is the *reconstruction* of stimuli from an observed connectivity matrix ***J***, which is different from the *retrieval* problem [20, 39–41], where we ask whether the patterns ***X*** are stable fixed points of the dynamics of the network, Eq (1). We will discuss the feasibility of reconstructing patterns from the network’s dynamics at the end of the paper.

### Solving the inference problem using statistical physics

Our aim is to reconstruct the patterns X* that were used to create the connectivity matrix ***J*** of a Hopfield network using the connectivity structure (3). We will call the patterns X* the ground truth of the problem. Since the connectivity structure is stochastic, we formulate the pattern reconstruction as a probabilistic inference problem. We interpret the connectivity matrix of the network ***J*** as a noisy observation of the symmetric low-rank matrix ***W*** ∼ X*(X*)^⊤^, which was distorted by the transformation given by the rule (3). We can characterise the conditional probability distribution of a weight *J*_*ij*_ given *W*_*ij*_ as

$$\begin{array}{cc} P_{\text{out}}\left(J_{ij}=0|W_{ij}\right) & =\frac{1}{2}\text{erfc}(\frac{W_{ij}-\tau }{\sqrt{2}\nu }), \end{array}$$ (5a)

$$\begin{array}{cc} P_{\text{out}}\left(J_{ij}|W_{ij}\right) & =\frac{\text{exp}(-{\left(J_{ij}-W_{ij}+\tau \right)}^{2}/2\nu^{2})}{\sqrt{2\pi }\nu }\;\text{for}\;J_{ij}>0. \end{array}$$ (5b)

Reconstructing low-rank matrices from such noisy, distorted observations is a generic inference problem that appears in a lot of different applications, such as robust [42] and sparse [43–45] PCA, Gaussian mixture clustering [46], and community detection in dense networks [47, 48], to name but a few. Low-rank matrices have also been used extensively in neuroscience to model recurrent connectivity [26–29]. The advantage of a Bayesian approach in all these problems is that they allow for a principled and transparent integration of knowledge about the problem into the inference process, for example through the choice of prior distribution and output channel. In the following, we will assume that we know the hyper-parameters *ν* and *τ* that were used to generate the connectivity matrix ***J***. Expectation maximisation and related techniques seem natural candidates to extend our approach to cases where we would need to learn these hyper-parameters (see Discussion).

Here, we adopt a Bayesian approach [49] to the inference of the patterns given the connectivity ***J***. This means that we will consider our reconstruction of the patterns as a random variable ***X***, whose posterior distribution *p*(***X***|***J***) given the connectivity matrix is given by Bayes’ theorem:

$$\begin{array}{c} P\left(X|J\right)=\frac{1}{Z(J)}\prod_{i}^{N}P_{X}\left(x_{i}\right)\prod_{j>i}^{N}P_{\text{out}}\left(J_{ij}|W_{ij}\right). \end{array}$$ (6)

where we introduced the shorthand $x_{i}=X_{:,i}\in R^{P}$ for the *i*th column of ***X***. We assume that patterns are uncorrelated from each other and that we know the *a priori* distribution *P*_***X***_(***X***) over patterns that were stored in the network. This distribution could for example reflect the fact that we know that the memories stored in the network are binary, encoding whether a given neuron is firing or not, or that we have an idea of the probability that any given neuron is firing in a given memory.

Note that we are phrasing the problem of reconstructing the memories here as the problem of reconstructing the columns of the matrix ***X***, or equivalently, the tuning curves of each neuron. Eventually, we are interested the whole matrix ***X***, so it doesn’t matter whether we reconstruct all its columns or all its rows. However, it is more convenient from an algorithmic point of view to work with the columns, which is the approach we will adopt here. The marginals of the rows of this distribution, which are *N*-dimensional, provide the best estimate of the patterns that can be performed [49].

Evaluating the high-dimensional integral to obtain the marginals exactly is an intractable problem. Instead, here we exploit the formal analogy between the posterior distribution (6) and certain probability distributions that arise in statistical physics to derive an algorithm called “approximate message passing” [50], which performs approximate Bayesian inference of the memories stored in the network. Furthermore, we will demonstrate that techniques from statistical physics, in particular a tool called “state evolution” (SE), can be used to analyse the behaviour and the performance of this algorithm in quite some detail. Approximate message passing algorithms can be understood as a variant of belief propagation, a general algorithm for inference in graphical models that is usually credited to Pearl [51].

## Results

### A Bayesian algorithm for pattern reconstruction

The inference problem considered here, where we aim to recover a symmetric low-rank matrix from noisy observations, can be solved using a class of approximate message passing (AMP) algorithms for low-rank matrix factorisation called Low-RAMP. It was derived by Lesieur et al. [52], building on previous works [44, 53, 54] that provided AMP algorithms for particular instances of low-rank matrix factorisation problems. Low-RAMP is an iterative algorithm that produces estimates for the mean ${\hat{x}}_{i}$ of the marginal distribution of *p*(***x***_*i*_) and their covariance matrix *σ*_*i*_, where ***x***_*i*_ is in general the *i*th column of the low-rank matrix ***X*** that we are estimating by evaluating the posterior distribution (6). In the present case, ${\hat{x}}_{i}$ is the mean of the estimated ‘tuning curve’ of the *i*th neuron (see above). Using this framework, we will derive variants of the algorithm for the pattern reconstruction problem outlined in the previous section. We present the algorithm in detail in the Methods section.

We also provide a reference implementation of Low-RAMP for symmetric and bipartite matrix factorisation problems applicable to a number of different problems. It is designed to be easily extendable to other problems and also provides a number of further utility functions. All the results in this paper can be reproduced using this code.

### State evolution

The AMP algorithm has the distinctive advantage over other algorithms, such as Monte Carlo methods, that its behaviour in the limit *N* → ∞ for separable prior on the X*, random i.i.d. noise *ζij*, and number of patterns *P* = *O*(1), can be tracked exactly and at all times using the “state evolution” technique [50, 55]. The roots of this method go back to ideas originally introduced in physics to deal with a class of disordered systems called glasses [56, 57]. For the low-rank matrix factorisation problems we consider here, state evolution was derived and analysed in detail by Lesieur et al. [52], building on previous works that derived and analysed state evolution for other specific problems [44, 45, 53]. The last few years in particular have seen a surge of interest in using state evolution to understand the properties of approximate Bayesian inference algorithms for a number of problems [58].

Since we are adopting a probabilistic approach to estimating the patterns, we will call the reconstruction of the patterns the mean of the posterior distribution, which we denote by a hat: ${\hat{x}}_{i}$. Our goal is to track the mean-squared error mse_*X*_ of the reconstruction ${\hat{x}}_{i}^{t}$ of the true signal $x_{i}^{*}$ after *t* steps of the algorithm,

$$\begin{array}{c} \text{mse}\left(t\right)\equiv \frac{1}{N}\sum_{i}^{N}\left|\right|{\hat{x}}_{i}^{t}-x_{i}^{*}{\left|\right|}_{2}^{2}, \end{array}$$ (7)

where $\left|\right|\cdot {\left|\right|}_{2}^{2}$ denotes the Euclidean norm of a vector. The mse can be expressed in terms of a single matrix-valued parameter defined as

$$\begin{array}{c} M^{t}\equiv \frac{1}{N}\sum_{i}^{N}{\hat{x}}_{i}^{t}x_{i}^{*,⊤}\in R^{P\times P}, \end{array}$$ (8)

such that $\text{mse}\left(t\right)=\text{Tr}[\langle x_{0}x_{0}^{⊤}\rangle -M^{t}]$. Here and throughout this paper we write averages with respect to the prior distribution *p*_*X*_(***x***) of the corresponding model as 〈⋅〉. We write ***x***_0_ with the subscript to underline that the random variable ***x***_0_ is not a column of the matrix ***X*** that we’re trying to evaluate; instead, it is a variable that is drawn from the prior and averaged over.

Now the goal is to find an update equation for the order parameter *M*^*t*^ that mirrors the progress of the algorithm. This update equation is the state evolution equation [50, 55]. Remarkably, from [59] we see that the two constants defining our problem, *τ* and *ν*, do not appear explicitly in the state evolution equations. Instead, the behaviour of the algorithm—and hence its performance—only depends on an effective signal-to-noise ratio (SNR) of the problem, which is a function of the threshold *τ* and noise variance *ν* utilised in the connectivity structure (3). Formally, it can be expressed as the inverse of the Fisher score matrix [60] of the generative model we use to describe how the network is connected (5a) and (5b), evaluated at $W_{ij}=x_{i}x_{j}^{⊤}/\sqrt{N}=0$:

$$\begin{array}{cc} \frac{1}{\Delta } & \equiv E_{P_{\text{out}}\left(J|w=0\right)}{\left(\frac{\partial \;\text{ln}\;P_{\text{out}}\left(J|w\right)}{\partial w}\right)}_{J,w=0}^{2} \end{array}$$ (9)

$$\begin{array}{cc} & =\frac{\tau e^{-\tau^{2}/2\nu^{2}}}{\sqrt{2\pi }\nu^{3}}+\frac{e^{-\tau^{2}/\nu^{2}}}{\pi \nu^{2}\text{erfc}(-\tau /\sqrt{2}\nu )}+\frac{1}{2\nu^{2}}\text{erfc}(\frac{\tau }{\sqrt{2}\nu }). \end{array}$$ (10)

Here and throughout, E denotes the expectation over the random variables. In fact, on the level of the algorithm, everything about the output channel (5) can be summarised in this single, scalar quantity Δ. This remarkable universality of the state evolution and hence the AMP algorithm with respect to the output channel was first observed in [59] and dubbed “channel universality”.

State evolution provides an update equation for the order parameter *M*^*t*^ that mirrors the progress of the algorithm. We first define an auxiliary function

$$\begin{array}{c} f\left(A,b\right)=\frac{1}{Z(A,b)}\sum_{x\in X^{P}}x\;p_{X}\left(x\right)\text{exp}(bx-\frac{1}{2}x^{⊤}Ax). \end{array}$$ (11)

where $A\in R^{P\times P}$ and $b\in R^{P}$. If ***A*** = 0 and ***b*** = 0, this function would compute the average over the prior distribution *p*_*X*_(***x***). Instead, ***b*** and ***A*** are estimated from the data (see the algorithm for details) so *f* computes an average over a distribution that contains the prior and a data-dependent part. This structure reflects the Gaussian approximation of the posterior density that we apply here, or more broadly speaking the interplay between prior information and data-dependent likelihood that is typical of Bayesian inference. Consequently, Z(A,b)=∑x∈XPpX(x)exp(bx-12x⊤Ax) is a normalisation factor. The update equation for the order parameter *M*^*t*^ can be written using this auxiliary function for all the cases considered in this paper; it reads [52]

$$\begin{array}{c} M^{t+1}=\underset{x_{0},z}{E}[f(\frac{M^{t}}{\Delta },\frac{M^{t}}{\Delta }x_{0}+\sqrt{\frac{M^{t}}{\Delta }}z)x_{0}^{⊤}] \end{array}$$ (12)

where ***z*** is a *P*-dimensional vector of Gaussian random variables with mean zero and variance 1. The average over ***x***_0_ is taken with respect to the prior distribution *p*_*X*_(***x***), as discussed above.

So to summarise, statistical physics gives us an algorithm to perform approximate inference of the patterns and the state evolution Eq (12) allows us to track the behaviour of the algorithm over time. We can thus analyse the performance of the algorithm in high-dimensional inference by studying the fixed points of the low-dimensional state-evolution (12). This is the key idea behind this approach, and we will now demonstrate the usefulness of this machinery by applying it to several specific cases.

### Reconstructing binary patterns

As a first application of the algorithm and the analysis tools outlined so far, we consider the reconstruction of a set of binary patterns, X={±1}. We will assume that both positive and negative values are equiprobable and that the components of a pattern vector are independent of each other, so the prior on a column of the matrix of stored patterns, ***x***_*i*_, is simply

$$\begin{array}{c} p_{X}\left(x_{i}\right)=\prod_{j}^{P}p_{x}\left(X_{ij}\right)=\frac{1}{2^{P}}. \end{array}$$ (13)

#### A single pattern (*P* = 1)

It is instructive and helpful for the following discussions to first consider the case where *P* = 1, *i.e.* there is only a single pattern stored in the network that we are trying to recover from ***J***. The threshold function for the model then becomes *f*(*A*, *B*) = tanh(*B*), with B∈R, and the state evolution for the now scalar parameter *m*^*t*^ simplifies to

$$\begin{array}{c} m^{t+1}=\underset{z}{E}\;\text{tanh}(\frac{m^{t}}{\Delta }+\sqrt{\frac{m^{t}}{\Delta }}z) \end{array}$$ (14)

where *w* is a scalar Gaussian random variable with zero mean and unit variance.

**Fig 1:**
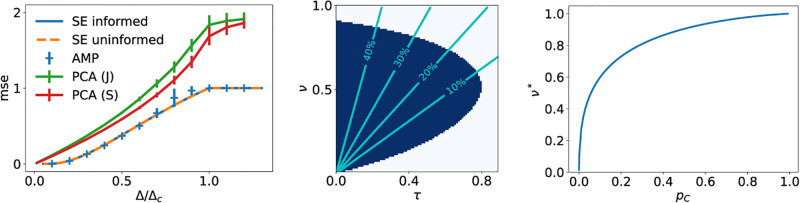
(*Left*) Reconstruction and performance of the message-passing algorithm for binary patterns. We plot the mse (7) obtained by the AMP algorithm (32) as a function of the effective noise Δ (9) (blue crosses). We plot the performance of the algorithm starting from random (15) and informed (16) initialisations. Solid lines depict the prediction obtained from iterating the state evolution Eq (14). Having Δ/Δ_*c*_ > 1 corresponds to the white region in the phase diagram on the right. We also plot the mse of the reconstruction obtained by applying PCA to the weight matrix ***J*** and to the Fisher matrix ***S*** (31) (green and red, resp.) *Parameters:* *τ* = 0. *N* = 5000 for AMP, *N* = 20000 for PCA. *(Center)* **Phase diagram for the rectified Hopfield channel with** *P* = 1. We plot whether reconstruction of the patterns better than a random guess is easy (blue) or impossible (white) using the message-passing algorithm as a function of the constant threshold *τ* and the variance *ν* of the Gaussian noise appearing in the connectivity structure (3). The solid lines are the contours of the connection probability *p*_*C*_(*ν*, *τ*) (4). *(Right)* Critical noise ν* as a function of connection probability *p*_*C*_. We plot ν*, the largest variance of the additive Gaussian noise *ζ*_*ij*_ at which reconstruction remains possible, against the probability *p*_*C*_ (4) that any two neurons are connected.

We can now iterate the state evolution Eq (14) with a given noise level Δ(*ν*, *τ*) until convergence and then compute the mse corresponding to that fixed point. The fixed point we converge to reveals information about the performance of the AMP algorithm. We plot the results on the left-hand side of Fig 1 for the two different *initialisations* of the algorithm: in blue, we plot the mse obtained by iterating SE starting with an *random initialisation*

$$\begin{array}{c} m^{t=0}=0+\delta , \end{array}$$ (15)

where *δ* > 0 is a very small random number. The error obtained in this way is the one that is obtained by the AMP algorithm when initialised with a random draw from the prior distribution—in other words, a random guess for the patterns. This is confirmed by the blue crosses, which show the mean and standard deviation of the mse obtained from five independent runs of the algorithm on actual instances of the problem. The dashed orange line in Fig 1 shows the final mse obtained from an informed initialisation

$$\begin{array}{c} m^{t=0}=1-\delta , \end{array}$$ (16)

which would correspond to initialising the algorithm with the solution, i.e. ${\hat{x}}_{i}=x_{i}^{*}$.

In this model, we find that the AMP algorithm starting from a random guess performs just as well as the algorithm starting from the informed initialisation. This need not always be the case, and we will indeed find a different behaviour in the next sparse and skewed models we consider.

#### When is recovery possible?

We can see from the middle plot of Fig 1 that recovery of the memories from the connectivity ***J*** is not always possible; there exists a critical value for the effective noise Δ_*c*_ above which the mean-squared error of the solution obtained by the algorithm is the same as we would have obtained by making a random guess for the solution based on the prior distribution (13) alone, without looking at the data. We can calculate this critical noise level Δ_*c*_ using the state evolution (12). We can see from that equation that *m*^*t*^ = 0 is a trivial fixed point, in the sense that the mse corresponding to that fixed point is equal to the mse obtained by making a random guess. Expanding Eq (14) around this fixed point yields *m*^*t*+ 1^ = *m*^*t*^/Δ. There are hence two regimes for recovery, separated by a critical value

$$\begin{array}{c} \Delta_{c}=1 \end{array}$$ (17)

of the effective noise (9). If Δ > Δ_*c*_, the uniform fixed point is stable and recovery is impossible. On the other hand, for Δ < Δ_*c*_, the uniform fixed point is unstable and hence AMP returns an estimate for the patterns that has an mse that is lower than random guessing. The phase diagram in the middle of Fig 1 delineates the easy and the impossible phase for the rectified Hopfield channel with symmetric prior (13). While there could be in principle other fixed points of the state evolution equations for other priors and channels [52], it is always one of the fixed points that is reached from either the informed or the uninformed initialisation that describes the behaviour of the algorithm.

At first sight, the impact of the additive Gaussian noise *ζ*_*ij*_ on the phase diagram in Fig 1 appears counter-intuitive. If we fix the threshold to, say, *τ* = 0.5, reconstruction is impossible for small variances *ν* of *ζ*_*ij*_. As we increase *ν*, i.e. as we add *more* noise to the system, recovery becomes possible. The key to understanding this behaviour is that for a single stimulus *P* = 1, a weight in the network will have one of two possible values which are symmetric around the origin, $W_{ij}=\pm^{1}/\sqrt{N}$. By applying the rectification, for any cut-off *τ* > *W*_*ij*_ the resulting weight matrix ***J*** without additive noise is trivially zero and no recovery is possible. We can only hope to detect something when an added noise *ζ*_*ij*_ pushes the value of the weight before rectification above the cut-off. Recovery then becomes possible if the added noise is large enough that the weight without noise is larger than the cut-off *a* + *ζ*_*ij*_ > *τ*, while remaining small enough that it’s significantly more likely that the noise-less weight is positive than negative. As the noise variance increases even further, its detrimental effects dominate, and recovery becomes impossible again. This mechanism is reminiscent of stochastic resonance (SR), a mechanism where a weak signal is amplified by the presence of noise. Indeed, our problem contains the three ingredients for SR (e.g. [61]): A threshold mechanism, given by the rectification in the connectivity structure: A weak signal (the stored patterns); and a noise term, *ζ*.

As already mentioned, when noise is too large recovery becomes impossible. We show on the right of Fig 1 the critical variance of *ζ*_*ij*_ above which reconstruction becomes impossible, ν*, as a function of the connection probability *p*_*C*_, given by Eq (4). This plot can be obtained by solving, for a given value *p*_*C*_ = *c*, the two-dimensional system

$$\begin{array}{cc} \Delta & =1 \end{array}$$ (18)

$$\begin{array}{cc} p_{C} & =c \end{array}$$ (19)

for (*τ*, *ν*). As expected, the critical variance increases with the connection probability, and it goes to zero as the connection probability goes to zero.

#### Comparison to principal component analysis (PCA)

Principal component analysis (PCA) is another method to reconstruct the stored patterns from the network connectivity. PCA and other spectral methods have some advantages: they are non-parametric, and their implementation in the case of a single pattern is straightforward: the PCA prediction for the stored pattern is simply the leading eigenvector of ***J***. We plot the mean-squared error (7) of this estimate with the green line on the left of Fig 1, where we see that the reconstructing error of PCA is larger than the one of AMP, especially for large values of the noise. This is also borne out by theory: the reconstruction mean-squared error of PCA can be shown to be strictly larger than the AMP estimate, since the latter is the Bayes-optimal predictor [52].

An alternative PCA algorithm can be found by linearising the AMP equations around the trivial fixed point $\hat{x}=0$ [58, 62]. This linearisation yields an equation that can be interpreted as PCA applied to the Fisher matrix ***S*** (31) instead of ***J***. Since the Fisher matrix depends on the generative model for the data when deriving the message-passing equations, looking at its leading eigenvector offers a spectral algorithm that is more adapted to the problem at hand. Indeed, we find that its error (red line in Fig 1) is slightly lower than the error obtained from PCA on the weights directly. In either case, the performance of PCA is worse than that of AMP.

The large value of the PCA error compared to the AMP error at large noise levels in Fig 1 reveals a fundamental weakness of PCA: even at noise levels above the critical noise Δ_*c*_, where no reconstruction is possible for any algorithm, PCA can be applied and will return a prediction—there is no concept of uncertainty in PCA. Hence the mse of PCA tends to a constant as the noise increases and the leading eigenvector of ***J*** is just a random vector; for the Hopfield prior and when rescaling the eigenvectors to have the same length as draws from the Hopfield prior, this constant is 2. AMP on the other hand returns a vector full of zeros if Δ > Δ_*c*_ (and the prior has an average of 0, as is the case for all the priors we consider). AMP thus expresses its uncertainty about the planted pattern, yielding an mse = 1 for inputs with *x*_*i*_ = ±1. The advantage of the Bayesian approach is thus that it prevents over-confident predictions in the high noise regime.

The weaker performance of PCA compared to AMP is due to the fact that spectral methods do not not offer a natural way to incorporate the prior knowledge we have about the structure and distribution of stimuli into the recovery algorithm. The Bayesian framework incorporates this domain knowledge in a transparent way through the generative model of the stored patterns *p*_*X*_(***x***). We will see that this creates an even larger performance gap for sparse patterns and patterns with low coding level.

#### Many patterns (*P* > 1)

For the general case of several patterns *P* > 1 with finite *P*, we can significantly simplify the state evolution by noticing that the matrix *M*^*t*^ will interpolate between a matrix full of zeros at time *t* = 0 and a suitably scaled identity matrix in the case of perfect recovery, *i.e.*

$$\begin{array}{c} M^{t}=m^{t}I_{P}/\Delta , \end{array}$$ (20)

where ***I***_*P*_ is the identity matrix in *P* dimensions. In other words, for uncorrelated patterns, the different input patterns do not interact during the reconstruction, and so the off-diagonal matrix elements remain zero in the case where we only store a few patterns and the connectivity structure remains low-rank. Once we overload the model by storing many more patterns, we would have non-zero off-diagonal elements, meaning that reconstructions converge to spurious patterns, for example linear combinations of the patterns. However, in this regime the state evolution derived above also breaks down. In this case, the threshold function becomes

$$\begin{array}{c} \left[{f(A,B)}_{k}\right]=\text{tanh}(\frac{m^{t}}{\Delta }x_{0,k}+\sqrt{\frac{m^{t}}{\Delta }}z_{k}) \end{array}$$ (21)

where *z*_*k*_ is again a standard Gaussian variable. Substituting into the state evolution gives an update equation for the parameter *m*^*t*^, namely

$$\begin{array}{c} m^{t+1}=\underset{z}{E}\;\text{tanh}(\frac{m^{t}}{\Delta }+\sqrt{\frac{m^{t}}{\Delta }}z) \end{array}$$ (22)

where *m*^*t*^ is the overlap parameter introduced above (20). This update has the same form as the state evolution in the *P* = 1 case, Eq (14). So we find, remarkably, that recovering *P* distinct patterns is exactly equivalent to recovering a single pattern *P* times in the thermodynamic limit where *N* → ∞ while the number of patterns is of order P∼O(1). This approximation will eventually break down in practical applications with finite network sizes, and we investigate the breaking point of this behaviour below.

Recovering many patterns with PCA poses an additional challenge. While it is easy to recover the leading rank-*P* subspace of the matrices ***J*** or ***S*** by simply computing the *P* leading eigenvectors, it is not clear how to recover the exact patterns from these eigenvectors, which can be any rotation of the input patterns due to the rotational symmetry of ***W***. This can be seen from the fact that the patterns X* could be multiplied by any rotational matrix ***O*** with $OO^{⊤}=I$ without changing the resulting weight matrix ***J***, see Eq (2). The best way to recover the exact stimuli from the principal components is thus not clear *a priori* (see [63]). Other problems require combining PCA with other methods, such as *k*-means or gradient descent. Since we have already seen that AMP outperforms PCA on binary patterns, and we will see that this gap only increase for the other types of patterns we will study below, we do not investigate further this direction.

#### A first summary

Before we turn to more complicated prior distributions over the patterns, let us briefly summarise our results so far. We derived an algorithm that can reconstruct patterns from the connectivity of recurrent network whose weights are obtained from the learning rule Eq (3). Whether or not the algorithm is successful in this reconstruction depends on the noise level *ν* and the threshold *τ*. These parameters can be combined into an effective noise parameter Δ (9), which determines the performance of the message-passing algorithm. The algorithm performs well in the reconstruction task, and beats non-parametric approaches like PCA by virtue of including prior information about the distribution of the patterns in a principled way.

### Sparse patterns

An interesting variation of the rectified Hopfield model is its sparse version, where only a fraction 0 ≤ *ρ* ≤ 1 of the components *x*_*ij*_ of a pattern ***x***_*i*_ are non-zero. The prior distribution then becomes

pX(xi)=∏j=1Ppx(xij)=∏j=1P[(1-ρ)δ(xij)+ρ2[δ(xij-1)+δ(xij+1)]], (23)

where *δ*(⋅) is the Kronecker delta. This prior has mean 〈*x*〉 = 0_*p*_, where 0_*p*_ is a vector of *p* zeros, and covariance 〈*xx*^⊤^〉 = *ρ**I***_*p*_. We emphasize again that these patterns represent a deviation of neuronal activity from its mean—in this case ±1 represents an increase/decrease activity, while 0 represents no change in activity in a given pattern. The state evolution will interpolate between an order parameter that is all zeros for an estimator that is drawn from the prior distribution and completely uncorrelated with the ground truth, and *M* = *ρ**I***_*P*_ for perfect reconstruction. We delegate the mathematical details to the Methods section, and focus here on the performance of the algorithm.

#### Performance of message-passing

**Fig 2:**
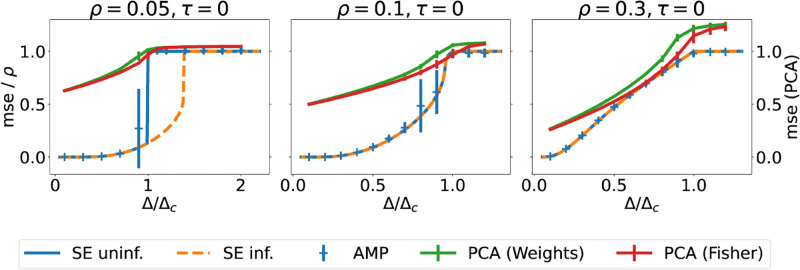
Reconstructing *sparse*, binary patterns using message passing algorithms and PCA. We plot the mse per pattern obtained by the AMP algorithm, Eq (32), as a function of the effective noise Δ (9), for random (15) and informed (16) initialisations. Lines depict the result of the state evolution, while crosses denote the performance of the AMP algorithm on an instance of the problem. While AMP performs the same starting from both initialisations for *ρ* = 0.1 and *ρ* = 0.3, there is a gap in performance for *ρ* = 0.05, which might hint at the existence of a hard phase (see main text). We also plot the mse of the reconstruction obtained by applying PCA to the weight matrix ***J*** and to the Fisher matrix ***S*** (31) (green and red, resp.) *Parameters:* *τ* = 0. *N* = 2000 for AMP, *N* = 20000 for PCA.

We first note that the critical noise level above which AMP will not be able to recover the stored pattern better than a random guess in the sparse reconstruction problem is Δ_*c*_ = *ρ*^2^. In that sense, the critical noise is a property of the AMP algorithm, and its value can again be obtained by linearising the state evolution Eq (36) around its trivial fixed point *m*^*t*^ = 0. However, it is generally believed that no other algorithm is able to recover the patterns above this noise level, which would make this threshold a property of the model rather than the algorithm; we’ll come back to this point in the next paragraph. In any case, the decrease of Δ_*c*_ with *ρ* means that the reconstruction performance at fixed *ν*, *τ* and hence Δ decreases with pattern sparsity. However, so too does the difficulty of the problem. If we normalise the noise level by the critical noise, *i.e.* if we plot the reconstruction error as a function of the Δ/Δ_*c*_ as we do in Fig 2, we see that a large fraction *ρ* of non-zero components *X*_*ij*_ leads to better reconstruction at small noise levels.

For *ρ* = 0.1 and *ρ* = 0.3, the performance plots in Fig 2 resemble the results obtained for the symmetric Hopfield model overall: Reconstruction is possible if the effective noise level is below the critical noise level. For these two values of the sparsity, the mse of the estimate returned by AMP (blue dashed line) matches the mse obtained from state evolution when starting from the informed initialisation (orange). However, the reconstruction errors predicted by state evolution for informed and random initial conditions *disagree* at smaller sparsity. For *ρ* = 0.05 (left), there is a range of effective noise levels Δ for which the performance of AMP does not match the performance predicted by state evolution starting from informed initial conditions. We note that this could be the signature of a so-called *hard phase*, where a better-than-chance reconstruction of the pattern is information-theoretically possible – i.e. there is some trace of the stored pattern in the connectivity—but AMP is not able to extract it. However, it is important to emphasise that AMP performs sub-optimally with respect to the amount of information that is in the connectivity, but not with respect to the performance of any other known algorithm. In other words, while AMP does not exploit all the information that is in the connectivity, it is broadly believed in theoretical computer science that *no algorithm* can reconstruct the patterns with non-trivial error at this level of noise in polynomial time, if this is indeed a hard phase of the reconstruction problem. PCA for example is also not able to reconstruct any trace of the pattern at this noise level (see below). We refer the interested reader to Refs. [58, 64] for recent reviews on the topic, with many other examples of problems that exhibit such a hard phase or computational-to-statistical gap, as it is also sometimes called in the literature.

#### Performance of PCA

Fig 2 also shows the reconstruction error of PCA applied to either the weights (green) or the Fisher matrix (red). Note that while the error of AMP is rescaled by *ρ*, we plot the reconstruction of PCA without rescaling (which is why we have a second *y*-axis in all three plots). In other words, the reconstruction error of AMP is multiplied by 20 in the left-most plot of Fig 2, and is still below that of PCA. We rescale the AMP error in this way to ensure that the errors are comparable for different values of the sparsity, since AMP returns a sparse estimate in all cases. PCA on the other hand is agnostic about the sparsity of the patterns, and returns a dense reconstruction regardless of the value of *ρ*. The PCA error at high noise thus scales as 1 + *ρ*. Again, we see that AMP outperforms PCA in terms of the reconstruction error, with the largest difference coming at lowest sparsity. This is to be expected, since the AMP algorithm can take information about the prior into account.

### Reconstructing patterns with low coding level

As a third and final example, we consider the reconstruction of patterns with low coding level. For this, we will draw patterns from the prior distribution

$$\begin{array}{c} p_{X}\left(x_{i}\right)=\prod_{j=1}^{P}p_{x}\left(x_{ij}\right)=\prod_{j=1}^{P}[\left(1-\rho \right)\delta \left(x_{ij}+\rho \right)+\rho \delta \left(x_{ij}-\left(1-\rho \right)\right)] \end{array}$$ (24)

which is related to models proposed first by Tsodyks and Feigel’man [21, 65], that considered the storage of binary (0,1) patterns of activity (where 0 means inactive and 1 active), with a ‘coding level’ *ρ* (probability that a neuron is active in a given pattern). The motivation for studying the sparse *ρ* ≪ 1 limit is that the activity in brain structures involved in memory is typically sparse—for instance, *ρ* has been estimated to be on the order of 0.01 in animals ranging from rodents to humans [66, 67]. As in the previous cases, the patterns correspond to deviations of activity from its mean, i.e. (1 − *ρ*, − *ρ*) instead of (1,0). This model has mean zero and a covariance matrix *ρ*(1 − *ρ*)***I***_*P*_. We still use the channel corresponding to the connectivity structure of the rectified Hopfield model (3), so while the Fisher score matrix ***S*** stays the same, we have a new threshold function and hence a new state evolution, which we derive in *Methods*.

**Fig 3:**
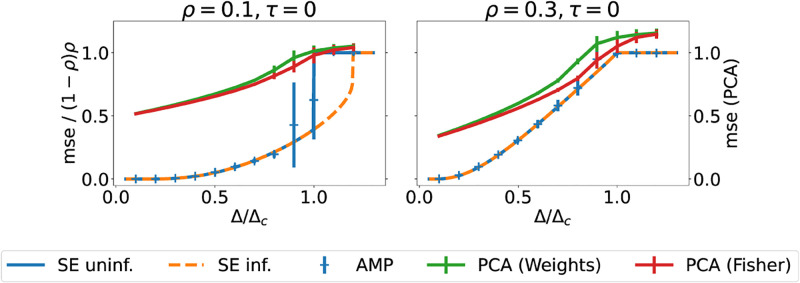
Performance of various algorithms when reconstructing a pattern with low coding level following Tsodyks’ prior. *(Top)* Same plot as Figs 1 and 2, for a single stored pattern drawn from Tsodyks’ prior (24). For *ρ* < 0.211, the performance of the algorithm with random initialisation (15) is different from the performance with informed initialisation (16), which might be the signature of a hard phase. *Parameters:* *τ* = 0, *N* = 5000 for AMP, *N* = 20000 for PCA.

Here, we plot the performance of the algorithm together with the results of iterating the state evolution equation (*Methods*) for two different values of the coding level *ρ* in Fig 3. Note that we again rescale the reconstruction error of AMP by (1 − *ρ*)*ρ* to ensure comparability of the results for different values of the coding level *ρ*. The error curves for PCA (green and red) in Fig 3 on the other hand are not rescaled. We note that again, AMP outperforms PCA in terms of the reconstruction error, which for random guessing tends to 1 + *ρ*(1 − *ρ*) in the case of PCA.

#### The hardness of recovering patterns with low coding level

In this model too, we have a uniform fixed point with *m*^*t*^ = 0. Expanding around this fixed point yields the update equation

$$\begin{array}{c} m^{t+1}=\frac{{\left(\rho -1\right)}^{2}\rho^{2}}{\Delta }m^{t} \end{array}$$ (25)

so we find that the critical value of the noise where the uniform or uninformative fixed point becomes unstable in this model is Δ < Δ_*c*_ = (*ρ* − 1)^2^*ρ*^2^.

Recently, a closed-form sufficient criterion for the existence of a hard phase in models that have a prior with zero mean was derived in [52]; namely, a hard phase exists if the prior is “skewed” in the statistical sense, such that

$$\begin{array}{c} {\left\langle x^{3}\right\rangle }^{2}>2{\left\langle x^{2}\right\rangle }^{3} \end{array}$$ (26)

where the average is taken with respect to the prior distribution of this model, Eq (23). For the prior (24), this criterion predicts the existence of a first order phase transition and hence of a hard phase for $\rho >\rho_{c}=1/2-1/\sqrt{12}≃0.2113$ where we assume w.l.o.g. that *ρ* < 1/2. Note that this is a sufficient condition, and not a necessary one; in fact, for the sparse Hopfield prior, which is symmetric around zero and has 〈*x*^3^〉 = 0, we cannot calculate the critical value of *ρ* using Eq (26).

### Reconstructing even more patterns: How far can we go?

A natural question that arises for the algorithms we have derived is how many patterns we can reliably reconstruct. In practice, the bottleneck for reconstructing the patterns using AMP is computing the partition function of the Gaussian approximation of the posterior density of a column-vector ***x*** at every step of the algorithm (see the detailed explanation of the AMP algorithm in the Methods section):

$$\begin{array}{c} W\left(x;A,b\right)=\frac{1}{Z(A,b)}p_{X}\left(x\right)\text{exp}(\sum_{j}^{P}b_{j}x_{j}-\frac{1}{2}\sum_{j,k}^{P}x_{j}A_{jk}x_{k}). \end{array}$$ (27)

Evaluating the mean and the variance of this distribution even for the simple Hopfield prior (13) requires summing 2^*P*^ terms, so the computational cost is exponential in the number of patterns stored. We can circumvent this bottleneck by computing the function *W*(***x***;***A***, ***b***) using a mean-field approximation [68], which was originally proposed in [69].

#### The mean-field approximation

**Fig 4:**
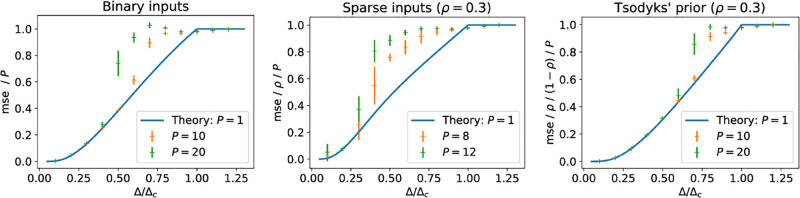
MSE from reconstructing with the mean-field prior approximation. We plot the final mse obtained by the AMP algorithm (no damping), using a mean-field approximation to compute the threshold function. The solid line is the mse predicted by iterating the state evolution for the scalar variable, Eq (14). We choose *N* = 5000 and set *τ* = 0.

We thus approximate the posterior distribution by a factorised distribution, replacing the full covariance matrix ***A*** with a vector ***a*** that contains only the variance of the *j* = 1, …, *P* elements of ***x***,

$$\begin{array}{c} \tilde{W}\left(x;\tilde{a},\tilde{b}\right)=\prod_{j}^{P}\frac{1}{\tilde{Z}\left({\tilde{a}}_{j},{\tilde{b}}_{j}\right)}p_{X}\left(x_{j}\right)\text{exp}({\tilde{b}}_{j}x_{j}-\frac{1}{2}{\tilde{a}}_{j}x_{j}^{2}). \end{array}$$ (28)

where we use the tilde to denote mean-field quantities. We have implemented mean-field approximations for the models discussed thus far and we show the performance of AMP with this approximation for the three models discussed so far in Fig 4 with the state evolution prediction for the reconstruction of a single stored pattern *P* = 1 without the mean-field approximation. The picture that emerges is similar for all three models studied here: the algorithm with the mean-field approximation is able to reconstruct the stored patterns just as well as if it was looking at the reconstruction of a single pattern up to a certain noise level, beyond which performance quickly deteriorates. Intuitively, the cross-talk between the stored patterns introduces an additional source of noise for the reconstruction, which leads to failure to reconstruct the stored patterns at lower Δ than in the case *P* = 1.

#### Scaling of the critical number of stored patterns

Throughout this paper, we have relied on the assumption that the matrix ***J*** is approximately low-rank, in the sense that its eigenvalues can be separated into large bulk of eigenvalues, from a which only P∼O(1) eigenvalues corresponding to the stored patterns detach. The mean-field approximation that we just introduced makes it now computationally feasible to run the reconstruction algorithm even with a large number of patterns. This raises an important practical question: for which number of patterns does the algorithm based on the low-rank approximation break down?

**Fig 5:**
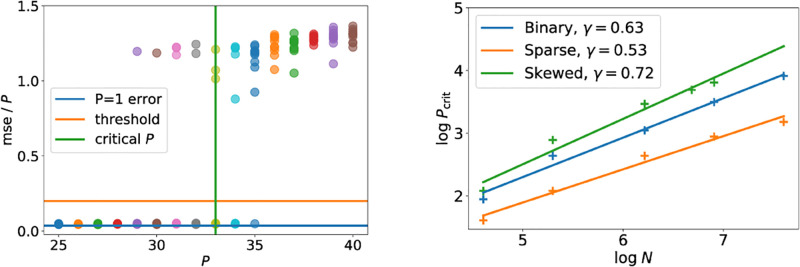
Scaling of the critical number of patterns that can reliably be reconstructed with mean-field message passing. *(Left)* For binary patterns, we plot the final reconstruction error of twenty instances of the mean-field message-passing algorithm as a function of the number of stimuli stored *P*. We define the critical number of patterns that can be reconstructed as the largest *P* at which more than half the runs yield an mse better than a threshold value (here, 20% of the trivial mse, i.e. 0.2). *Parameters:* *N* = 1000, Δ = 0.2Δ_*C*_, *τ* = 0. *(Right)* We plot the highest number of patterns *P*_max_ that could be reconstructed with an error below 20% of the trivial error in at least 50% of cases. For all three models considered, we show experimental results (crosses) and the exponent of a power law fit *P*_max_ ∼ *N*^*γ*^. In all cases, *τ* = 0 and the noise level Δ was 20% of the critical noise level Δ_*c*_ where reconstruction becomes impossible. *ρ* = 0.3 for both the sparse Hopfield and Tsodyk’s prior.

We investigate this question in all three models numerically as follows. We fixed the noise level Δ in all experiments at 20% of the critical noise level beyond which it is information-theoretically impossible to weakly reconstruct the stored patterns. We then ran twenty reconstruction experiments with *N* = 1000 for each value of *P*; the final mse for each run is shown with a dot on the left of Fig 5. For *P* = 25, all twenty runs gave an mse well below the threshold; in fact, the reconstruction error per pattern mse/*P* is as low as if we had stored only a single pattern in the network (blue line). As we increase the number of patterns *P*, the first time the algorithm is not able to recover all patterns is for *P* = 29 patterns. For *P* = 36 patterns, the algorithm did not reconstruct the patterns with an error better than chance a single time. Given the clear separation between successful runs with low error and unsuccessful runs with an error that is essentially random guessing, we set the threshold for the reconstruction mse below which we consider the algorithm successful at 20% of the trivial mse obtained by random guessing (orange line). At *P* = 34, the algorithm fails to achieve an mse below the threshold in more than 50% of the cases. We define the critical number of stored patterns *P*_crit_ as the largest number of patterns that can be reconstructed with an mse below the threshold in at least 50% of the runs, so *P*_crit_ = 33 in this case. On the right, we show the values of *P*_crit_ for all three models (binary, sparse and skewed) as a function of *N*. In each case, we find that *P*_crit_ ∼ *N*^*γ*^, with the exponent *γ* between *γ* ≈ 0.5 for sparse patterns and *γ* ≈ 0.7 for patterns from Tsodyk’s prior. The exact values of the exponents will depend on the choices of the threshold and especially the noise level. While the separation between successful and unsuccessful runs allows for various error thresholds without changing the results, the exponents are more sensitive to the noise level, since higher noise levels induce higher fluctuations in the reconstruction errors closer to the critical noise (cf. Fig 4).

## Discussion

We have derived the conditions under which it is possible to reconstruct the patterns stored in a recurrent Hopfield-type network from knowledge of the connections alone, in a case where those weights are obtained by a learning rule which is a noisy, non-linear version of the classic Hebbian rule. We have implemented and provided practical algorithms to do so: a Bayesian approach based on message passing, and classic non-parametric approaches such as PCA on the weights and on the Fisher score matrix. The message-passing algorithm offer a principled way to reconstruct the patterns using prior information about the prior of the inputs, while the spectral methods are robust and easy-to-implement, but fail to take this extra information into account.

The performance of the algorithms can be captured by an effective noise level, which takes both the synaptic noise and the thresholding of the learning rule into account. We found that the message-passing algorithm beats the PCA reconstruction across noise levels, which is due to the principled way in which the message-passing algorithm can incorporated prior knowledge about the distribution of patterns.

While our theoretical results were obtained in the limit where the number of patterns stored is of order 1, while the number of neurons in the network tends to infinity, we have also explored a mean-field approximation that allowed us to study how the maximum number of patterns that can be reconstructed with some reliability scales with the pattern dimension. We now discuss some directions in which our work can be extended.

### The case of unknown generative model

We have assumed that the hyper-parameters of the problem, such as the number of stored stimuli *P* or the generative model of the stimuli *p*_*X*_(***x***), are known before reconstruction. However, it is possible to extend both the message-passing algorithm and its analysis to the case of unknown hyper-parameters. Within our framework, a natural approach to learn the values of these hyper-parameters is closely related to the well-known expectation maximisation algorithm [70]. A detailed study of expectation maximisation to obtain various hyper-parameters for message-passing algorithms was reported by Krzakala et al. [71] for the statistical estimation problem of “compressed sensing”, where one aims to reconstruct a signal from a number of measurements that is smaller than the number of unknowns. Another approach to deal with an unknown noise distribution was recently proposed by Montanari et al. [72] for the related problem of matrix denoising, where one aims to reconstruct an unknown, low-rank matrix $X\in R^{m\times n}$ from observations ***Y*** = ***X*** + ***W*** when ***W*** is a noise matrix with independent and identically distributed entries. Montanari et al. [72] propose an iterative approach to estimate the noise distribution from the observations ***Y***. An interesting direction for future work is to explore both strategies for the problem of reconstructing stored patterns in connectivity matrices.

From the point of view of the theoretical analysis, assuming the “wrong” rank in the algorithm in the sense that there is a mismatch between the number of patterns one wants to infer and the number of patterns stored makes the analysis much more involved. The message-passing algorithms can be modified to take this mismatch into account. While first steps towards a theoretical analysis and the development of modified message-passing have recently been made [73–75], we leave the exploration of this direction in the context of neuroscience to future work.

Finally, there could also be no (discernible) signal in the connectivity matrix, because the relative strength of the noise is too large, or because the connectivity is purely random. As we discussed above, AMP shows a clear advantage over PCA in this case: while AMP will return inputs which are close to 0, indicating that it didn’t find any structure in the data, PCA will always return the leading eigenvector, which in this case however is completely uninformative.

### Reconstructing an extensive number of patterns

Another important extension of our work would be the reconstruction of an extensive number of patterns from the connectivity ***J***, *i.e.* whether the regime P∼O(N) is accessible. This is essentially the problem of factorising a large matrix with extensive rank, which is also known as dictionary learning [76–78]. While we note that there have been promising signs of progress recently [79, 80], this remains a hard problem with implications that would go far beyond the application discussed here.

### Reconstructing the patterns from the dynamics

It may be possible to retrieve a pattern given a distorted or noisy version of it by initialising the network with this distorted pattern and running the network dynamics (1) until convergence. Another algorithm to reconstruct the patterns is then to run the network dynamics from different initial conditions until convergence, and to take the resulting fixed points as estimates of the patterns stored in the connectivity. However, this procedure will only yield stored patterns if the initial condition is correlated with one of the patterns stored in the network. It is thus more apt to speak about “pattern completion” in this context [49]. Here, we considered the more challenging problem where such partial observations of the patterns are not available, which would be the case if the only information available is the connectivity matrix.

It is still fair to wonder how well this algorithm could do when starting from random initial conditions. In the classical Hopfield model, where the weights are given by the Hebb rule (2), any initial condition of the dynamics that has non-zero macroscopic overlap with a stored pattern is guaranteed to converge to that pattern when the number of patterns is of order one. In a finite network, one then expects random initial conditions to converge to one of the stored patterns (depending on the random initial overlaps). While we study a learning rule that is different from Hebb’s rule, we note a classic result by Sompolinsky [35] that suggests that the dynamics of recurrent networks whose connections are a nonlinear, noisy version of the Hebbian weights (2) are largely unaffected by this change.

For a single pattern, iterating the dynamics resembles the power method [81] to compute the first principal component of a symmetric matrix, and the dynamical approach to reconstruction thus closely resembles the PCA algorithm that we analysed in the case *P* = 1. For more than one pattern, there is no guarantee however that *all* the patterns will be recovered. Moreover, the performance of this algorithm is expected to be strongly model-dependent: in more realistic models such as the Tsodyks-Feigel’man model [21, 65], the quiescent state, where all neurons are at zero, is stable, and convergence to one of the stored patterns will necessitate an initial overlap that is larger than some non-zero critical value. Recovering the patterns through the dynamics is not expected to work in this case.

Similarly, this strategy is also expected to fail when reconstructing an extensive number of patterns. In this case, we need a correlation between the initial condition and each pattern which is bounded away from zero, and obtaining such a correlation for all the patterns would require exponentially many trials.

### What information on synaptic weights does connectomic data provide?

EM reconstructions of neuronal circuits are of sufficiently high resolution that they can enable measurements of the volume of dendritic spines, the anatomical structures on which the vast majority of synaptic connections between pyramidal cells are formed (see e.g. [14]). The volume of dendritic spines has in turn be shown to be strongly correlated with synaptic strength, as measured by the amplitude of post-synaptic potentials triggered by pre-synaptic activity [82–84]. Noise in measurement of dendritic spines, and the lack of perfect correlation between volume and EPSP amplitudes, are two of the reasons (together with noise in the learning process) for the introduction of the noise matrix *ζ*_*ij*_ in our model.

### Towards more biologically realistic models

Our focus here was to analyse the simplest model where the problem of retrieving the patterns is mathematically well-posed, and neither trivial nor impossible to achieve. We now discuss various extensions of this model that could be addressed in future work.

#### Asymmetry in the learned component of the connectivity matrix

We have focused here on a symmetric connectivity matrix, for multiple reasons. First, multiple in vitro studies in both cortex and hippocampus have shown that local networks in these structures exhibit a significant degree of symmetry, as evidenced by a much higher probability of bidirectional connections in pairs of neurons, compared to a random directed Erdos-Renyi network [85–88], with the notable exception of rodent barrel cortex where no such overrepresentation exists [89]. In addition, synaptic plasticity in area CA3 of the hippocampus has been shown to be temporally symmetric [90]. One thus expects such synaptic plasticity rules to lead to connectivity matrices with a strong degree of symmetry in this area. In cortex, plasticity rules are temporally asymmetric as a function of the timing difference between spikes of pre and post-synaptic neurons. However, plasticity depends also strongly on firing rates of pre and post-synaptic neurons, and if dependence on firing rate dominates over the dependence on spike timing as has been suggested [91], then one also expects a strongly symmetric Hebbian component in connectivity matrices. Finally, a strong degree of symmetry is also consistent with the observation of persistent activity in multiple cortical areas in rodents [92] and primates [93–96]. Consistent with this idea, Inagaki et al [92] showed using optogenetic perturbations that the dynamics in area ALM of the mouse during a short-term memory task exhibit multiple characteristics of attractor dynamics. Of course, we do not expect connectivity matrices in all brain structures to be well captured by noisy symmetric matrix. In particular, networks storing temporal sequences [97–99] would need to contain a significant asymmetric learned component. Our methods would then need to be extended to asymmetric matrices. From the practical point of view, the application of the method proposed makes only sense when the reconstructed connectivity matrices exhibit a significant degree of symmetry.

#### Inhibition

Here, we have assumed inhibition is not involved in learning and simply provides a uniform inhibition to excitatory neurons, equivalent to setting a threshold for active neurons. This traditional view is consistent with the observation of high connection probabilities between a specific type of inhibitory neuron (PV positive interneurons) and pyramidal cells [100]. It is also consistent with the observation in hippocampus that inhibition is only weakly modulated by spatial location [32] (see however [101]). However, it has also been shown that synaptic connections involving inhibitory interneurons also exhibit plasticity [102], and it has been argued that plasticity of such connections could greatly expand storage capacity [34]. Our methods could be extended to the addition of inhibitory neurons, with a few caveats: estimate of the strength of such synapses might be more challenging, since synapses involving interneurons are formed directly on dendritic shafts and not on spines; Also, the connectivity matrix will then necessarily be asymmetric.

#### Memories stored with different strengths

In our model, as in most associative memory models, memories are stored with identical strengths. A straightforward extension of the model would be a model where memories have different embedding strengths. This class of models include ‘palimpsest’ models in which recently stored patterns gradually erase older patterns that are progressively forgotten [103–106]. Note that in this class of models, our method would be likely to infer only the most strongly embedded patterns, and memories that are on their way to becoming forgotten would not be likely to be inferred. From the point of view of message-passing algorithms, the gradual erasure would have to be modelled through the learning rule of the model, Eq (3), together with a prior over embedding strengths.

#### Distributions of synaptic weights

Our model leads to a truncated Gaussian distribution of non-negative synaptic weights. Distributions of experimentally recorded EPSP amplitudes [86] as well as spine volumes [107] have been fitted using log-normal distributions. Our method can easily be generalized to networks with arbitrary distributions of non-negative weights, by using a suitable non-linearity *Φ* in Eq (3) [34].

#### Binary Hebbian matrices

It has been proposed by some authors that synapses store information in a digital, not analogue fashion [35, 108, 109]. In this scenario, synapses have only a few stable states, and plasticity events correspond to transition between these states. The resulting model would then bear similarities with stochastic block models (SBMs) [110], where groups of neurons representing a particular stored memory would correspond to communities in SBMs. An important difference is that in the case of random patterns, there would be overlaps between these groups [111]. One could use similar methods as the ones proposed here to deal with such a scenario, since recovering communities in stochastic block models can be reformulated as a low-rank matrix factorisation problem, and is hence amenable to the same analysis techniques that we used here, see refs. [52, 64, 112] for examples of this rich literature.

#### Distributions of patterns

We have assumed that stored memories are random i.i.d. binary vectors. Responses of neurons to external inputs rarely follow a bimodal distributions, and can sometimes be better described by a unimodal lognormal distribution [113]. However, non-linearities associated with the synaptic plasticity rule could potentially binarize stored memories [24], which would then result in a model that is very similar to the model investigated here.

### Validation

An important question is how to validate the results of such an analysis. One possibility would be simulate the dynamics of a network whose connectivity matrix is set to be the reconstructed matrix, using the inferred patterns as initial conditions, to check that the dynamics converges to fixed point attractors that are close to the inferred patterns.

## Methods

### A formal analogy between inference problems and statistical physics

It may come as a surprise that statistical physics can be helpful in solving and analysing inference problems like the one considered here. The connection between the two becomes more transparent if we introduce the interaction *g*(⋅) ≡ ln *P*_out_(⋅) to rewrite the posterior as

$$\begin{array}{c} P\left(X|J\right)=\frac{1}{Z(J)}\prod_{i}^{N}P_{X}\left(x_{i}\right)\prod_{j>i}^{N}\text{exp}[g(J_{ij},W_{ij})]. \end{array}$$ (29)

This distribution describes the posterior density over estimates ***X***. However, it can also be interpreted as the Gibbs or Boltzmann distribution that describes the properties of complex, disordered systems such as glasses. This analogy can be leveraged by exploiting tools from the statistical physics of disordered systems to tackle the—hard—inference problem that is inferring the patterns from the connectivity ***J***. The key ideas behind the AMP algorithm that we discuss throughout this work and AMP algorithms in general first appeared in a paper by Thouless, Anderson and Palmer [114] that dealt with physical systems described by an equilibrium distribution of the type (29). State evolution techniques where first introduced for compressed sensing problems by Donoho, Maleki and Montanari [50] based on ideas from [55], but it too is based on ideas from statistical physics often referred to as the cavity method [56]. For a much more detailed on the links between statistical physics and inference problem, see [49, 58, 115].

### Approximate message passing for low-rank matrix reconstruction

We are now in a position to state the AMP algorithm for the factorisation of symmetric low-rank matrices that in this form was derived by Lesieur [52], building on the previous works deriving AMP-type algorithms for particular instances of this problem class [44, 53, 54]. We refer the interested reader to these papers for details on the derivation of this class of algorithms and their relation to belief propagation and spectral methods.

To describe the algorithm, we first define the Fisher score matrix as a transformation of the data matrix ***J*** given for the inference:

$$\begin{array}{c} S_{ij}\left(J_{ij}\right)\equiv {\frac{\partial \;\text{ln}\;P_{\text{out}}\left(J_{ij}|w\right)}{\partial w}|}_{w=0} \end{array}$$ (30)

For the channel corresponding to the rectified Hopfield model (3), we find

$$\begin{array}{c} S_{ij}\left(J_{ij}\right)=\{\begin{array}{cc} -\frac{2e^{-\tau^{2}/2\nu^{2}}}{\sqrt{2\pi }\;\nu \;\text{erfc}\;(-\tau /\sqrt{2}\nu )}, & J_{ij}=0\\ \frac{J_{ij}+\tau }{\nu^{2}} & \text{otherwise}. \end{array} \end{array}$$ (31)

Low-RAMP is an iterative algorithm: at every step *t*, it computes a new estimate of the mean ${\hat{x}}_{i}^{t+1}$ and the variance $\sigma_{i}^{t+1}$ as

$$\begin{array}{cc} {\hat{x}}_{i}^{t+1} & =f(A_{i}^{t},b_{i}^{t}) \end{array}$$ (32a)

$$\begin{array}{cc} \sigma_{i}^{t+1} & =\partial_{b}f\left(A_{i}^{t},b_{i}^{t}\right) \end{array}$$ (32b)

where the threshold function was defined in Eq (11) and is repeated here for convenience:

$$\begin{array}{c} f\left(A,b\right)=\frac{1}{Z(A,b)}\sum_{x\in X^{P}}x\;p_{X}\left(x\right)\text{exp}(bx-\frac{1}{2}x^{⊤}Ax). \end{array}$$ (33)

There exists a set of parameters $A_{i}\in R^{P\times P}$ and $b_{i}\in R^{P}$ for every marginal, which in turn are updated as

$$\begin{array}{cc} b_{i}^{t} & =\frac{1}{\sqrt{N}}\sum_{k}^{N}S_{ki}{\hat{x}}_{k}^{t}-(\frac{1}{N}\sum_{k}^{N}S_{ki}^{2}\sigma_{k}^{t}){\hat{x}}_{i}^{t-1} \end{array}$$ (34a)

$$\begin{array}{cc} A_{i}^{t} & =\frac{1}{N}\sum_{k}^{N}S_{ki}^{2}{\hat{x}}_{k}^{t}{\hat{x}}_{k}^{t,⊤} \end{array}$$ (34b)

To run the algorithm, we perform these steps:

1. Given the matrix ***J***, compute the Fisher score matrix ***S*** using Eq (31).
2. For all *i* = 1, …, *N*, initialise the parameters ***b***_*i*_ and ***A***_*i*_ such that all entries are zero. Initialise all estimators ${\hat{x}}_{i}^{t}$ with a random draw from the prior distribution *p*_*X*_(***x***) and set ${\hat{x}}_{i}^{t-1}$ to all zeros for the first step. (There is no need to initialise *σ*_*i*_).
3. Compute first the update to $A_{i}^{t}$ and $b_{i}^{t}$ following Eqs (34b) and (34a), then compute the new means ${\hat{x}}_{i}^{t+1}$ and their variance $\sigma_{i}^{t+1}$ using Eqs (32a) and (32b).
4. Repeat Step 3 until the squared difference between all ${\hat{x}}_{i}^{t}$ and ${\hat{x}}_{i}^{t+1}$ is smaller then some predefined threshold *ϵ*.

We also provide an implementation of this algorithm in a Python package that was the base of all the programs written for this paper.

### State evolution for reconstructing sparse patterns

We discussed in the main text that the sparse prior (23) has mean 〈*x*〉 = 0_*p*_ and covariance 〈*xx*^⊤^〉 = *ρ**I***_*p*_. The state evolution will interpolate between an order parameter that is all zeros at initialisation, and *M* = *ρ**I***_*P*_ for perfect reconstruction. We are thus motivated to use the ansatz *M*^*t*^ = *m*^*t*^***I***_*P*_. The threshold function then becomes

$$\begin{array}{c} \left[{f(A,B)}_{k}\right]=\frac{\rho e^{-A_{kk}/2}\text{sinh}\left(B_{k}\right)}{1+\rho [e^{-A_{kk}/2}\text{cosh}\left(B_{k}\right)-1]} \end{array}$$ (35)

Substituting this form into the SE update Eq (12) yields a closed update equation for the parameter *m*^*t*^,

$$\begin{array}{c} m^{t+1}=\underset{w}{E}\;\frac{\rho^{2}e^{-m^{t}/2\Delta }\text{sinh}\;(m^{t}/\Delta +\sqrt{m^{t}/\Delta }z)}{1+\rho \;[e^{-m^{t}/2\Delta }\text{cosh}\;(m^{t}/\Delta +\sqrt{m^{t}/\Delta }z)-1]}, \end{array}$$ (36)

where *z* is again a scalar Gaussian random variable with zero mean and unity variance, like in Eq (14). We can recover the state evolution for the symmetric rectified Hopfield model from Eq (36), in the limit *ρ* → 1.

### State evolution for reconstructing patterns with low coding level

For Tsodyk’s prior (24), we have a new threshold function (11) which reads

[f(A,B)k]=1-ρ-1-ρ1-ρ(1-eAkk(ρ-12)+Bk) (37)

The prior distribution (24) allows us to use the same ansatz for the magnetisation that we used above to analyse sparse patterns. Setting *M*^*t*^ = *m*^*t*^***I***_*P*_, we get the following update equation for the scalar order parameter *m*^*t*^ > 0:

mt+1=EW(ρ-1)2ρ2(emt/Δ-1)ewmt/Δ(ρewmt/Δ-(ρ-1)emt2Δ)(ρexp(2wΔmt+mt2Δ)-ρ+1). (38)
